# ^11^C-Labeling of a Flavanone Extracted from a South American Native Species for Evaluation of Its Interaction with GSK-3β

**DOI:** 10.3390/molecules30040874

**Published:** 2025-02-14

**Authors:** Maia Zeni, Maria Daniela Santi, Florencia Arredondo, Laura Reyes, Manuela Bentura, Diego Carvalho, Mariana Peralta, Maria Gabriela Ortega, Juan A. Abin-Carriquiry, Loreto Martínez-Gonzalez, Juan Pablo Gambini, Pablo Duarte, Ana Martinez, Ana Rey, Javier Giglio

**Affiliations:** 1 Graduate Program in Chemistry, Facultad de Química, Universidad de la República, General Flores 2124, Montevideo 11800, Uruguay; 2 Centro Uruguayo de Imagenología Molecular (CUDIM), Av. Ricaldoni 2010, Montevideo 11600, Uruguay; mdaniela0505@gmail.com (M.D.S.); florencia.arredondo@cudim.org (F.A.); laura.reyes@cudim.org (L.R.); manuela.bentura@cudim.org (M.B.); juan.gambini@cudim.org (J.P.G.); pablo.duarte@cudim.org (P.D.); javier.giglio@cudim.org (J.G.); 3 Instituto de Investigaciones Biológicas “Clemente Estable” (IIBCE), Av. Italia 3318, Montevideo 11600, Uruguay; diego.carvalhoalvarez@gmail.com (D.C.); aabin@pasteur.edu.uy (J.A.A.-C.); 4 Instituto Multidisciplinario de Biología Vegetal (IMBIV—CONICET), Av. Vélez Sarsfield 1611, Córdoba 5000, Argentina; maperalta@unc.edu.ar (M.P.); maria.gabriela.ortega@unc.edu.ar (M.G.O.); 5 Laboratorio de Farmacognosia, Departamento de Ciencias Farmacéuticas, Facultad de Ciencias Químicas, Universidad Nacional de Córdoba, Av. Medina Allende 1998, Córdoba 5000, Argentina; 6 Centro de Investigaciones Biológicas Margarita Salas, CSIC, Calle Ramiro Maetzu 9, 28040 Madrid, Spain; loretomg@cib.csic.es (L.M.-G.); ana.martinez@csic.es (A.M.); 7 Centro de Investigación Biomédica en Red Sobre Enfermedades Neurodegenerativas (CIBERNED), Instituto de Salud Carlos III, 28031 Madrid, Spain; 8 Área de Radioquímica, Facultad de Química, Universidad de la República, General Flores 2124, Montevideo 11800, Uruguay; arey@fq.edu.uy

**Keywords:** GSK-3β, Alzheimer’s disease, glabranin

## Abstract

Natural products play a crucial role in drug discovery, primarily due to their structural complexity. The prenylated flavanone glabranin ((S)-5,7-dihydroxy-8-(3-methylbut-2-en-1-yl)-2-phenylchroman-4-one), isolated from the Dalea elegans plant species, has demonstrated neuroprotective effects, attributed to its inhibition of GSK-3β, as per our previous in silico studies. Given the enzyme’s diverse functions and its potential as a target for neurodegenerative diseases, our group synthesized and evaluated an ^11^C-labeled derivative of glabranin. We present its in vitro biological activity, including IC_50_, neuronal uptake in Alzheimer’s-affected brain regions, key physicochemical properties, and animal studies. This study confirms [^11^C]FLA’s interaction with GSK-3β in vitro, highlighting the potential of radiotracers in bioactive compound research.

## 1. Introduction

Dementia is among the greatest global health crises of the 21st century. Presently, more than 50 million people are living with dementia worldwide, with this number estimated to triple to 152 million by 2050 as the world’s population grows older [1]. Alzheimer’s disease (AD) is the most common cause of dementia and is thought to account for 60–80% of the cases [2].

AD is indeed a neurodegenerative disorder primarily characterized by cognitive decline and progressive memory loss. At the molecular level are two key pathological conditions: the presence of neurofibrillary tangles (NFTs) and insoluble β-amyloid (Aβ) plaques [3]. Aβ is derived from the proteolytic cleavage of β-amyloid precursor protein, whereas NFTs comprise hyper-phosphorylated forms of the microtubule-associated protein tau.

Despite the progress made, the efficacy of current drugs to treat, delay, or block the progression of neurodegenerative diseases remains limited [4,5,6,7]. Therefore, the discovery and development of new molecules that can improve current treatments are of great importance.

Natural products have long been a cornerstone in drug discovery due to their structural diversity and ability to interact, in some cases, with biological targets with high specificity and affinity. These compounds, produced by organisms as adaptations to ecological and environmental pressures over billions of years, often exhibit a broad spectrum of biological activities, including antibacterial, antifungal, antioxidant, anticancer, anti-inflammatory, and neuroprotective properties, with minimal side effects [8,9,10,11].

Various bioactive molecules have been isolated from marine and terrestrial organisms, including amino acid-derived alkaloids, aromatic compounds, fatty acids, sphingolipids, lactones, alcohols, peptides, polyacetylenes, quinones, quinolones, sterols, terpenes, and terpenoids, among others. *Dalea* L. (Fabaceae) is a uniquely American genus with more than 172 species. Its habitat extends from central Argentina and Chile to the southwestern United States. Different flavonoids have been isolated from *Dalea Elegans*, mostly belonging to the group of prenylated flavanones, with diverse biological activities, such as antioxidants, toxic effects on mitochondria isolated from rat liver and human tumor cells, antimicrobials, and tyrosinase inhibitors [12].

**Figure 1:**
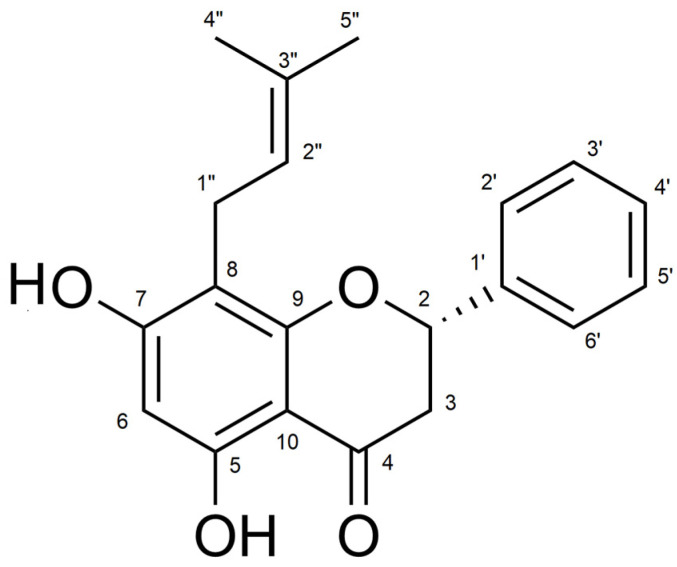
Chemical structure of (S)-5,7-dihydroxy-8-(3-methylbut-2-en-1-yl)-2-phenylchroman-4-one (glabranin).

In the study by Santi D. and colleagues [13], the neuroprotective activity of five prenylated flavanones isolated from *Dalea* species was evaluated in models of neurodegeneration in vitro. The flavanone (S)-5,7-dihydroxy-8-(3-methylbut-2-en-1-yl)-2-phenylchroman-4-one (Glabranin) (Figure 1) showed neuroprotective effects against oxidative stress-induced death in primary cultures of cerebellar granule neurons. In addition, an in silico screening of the most potent compounds was performed, looking for neuroprotective-related protein targets responsible for the effects of these flavanones, based on ligand similarity search, pharmacophore comparison, and reverse docking.

These in silico assessments suggest that their neuroprotective potential could potentially be mediated by interaction with glycogen synthase kinase-3 beta (GSK-3β), a serine/threonine kinase that plays a leading role in the cascade of events associated with AD. GSK-3β plays a central role in a wide variety of cellular processes concerned with coordinating catabolic and anabolic pathways [14,15,16,17]. Particular interest has been focused on GSK-3β in the context of neuronal functions since its activity is essential for normal neurodevelopment and its overactivity is implicated in psychiatric conditions, cognitive dysfunction, and neurodegeneration [15,18,19,20,21,22,23]. Furthermore, its overactivity accounts for protein tau hyperphosphorylation, increased β-amyloid production, and local plaque-associated microglial-mediated inflammatory responses, all of which are hallmark characteristics of AD.

Advances in imaging modalities such as positron emission tomography (PET) have encouraged its use for the evaluation of functional changes in brain pathologies. PET images provide information on pathological markers as well as pharmacokinetic properties to evaluate the absorption, distribution, metabolism, and excretion profiles of candidate drugs. Thus, PET imaging has become crucial for the characterization of new therapeutic agents.

Given the wide range of functions associated with GSK-3β, this enzyme has emerged as an interesting target for therapy and imaging in diverse diseases, especially AD. Considering all this information, our goal was to demonstrate the interaction between glabranin and GSK-3β as a putative target molecule involved in the neuroprotective effects of prenylated flavanones and to explore its potential as a GSK-3β marker. To accomplish these objectives, a radiolabeled glabranin derivative was prepared, and its physicochemical properties were determined to explore its suitability to cross biological membranes, in particular, the blood–brain barrier. In vivo and ex vivo animal biodistribution and imaging experiments were also performed to corroborate this capacity. In addition, this radiolabeled product was evaluated in vitro to determine its cellular and target specificity. Finally, the inhibitory activity towards GSK-3β (IC_50_) was determined.

## 2. Results

### 2.1. Chemistry

The glabranin derivative (*S*)-5-hidroxy-7-methoxy-8-(3-methylbut-2-en-1-yl)-2-phenylcroman-4-one (FLA) was synthesized using methyl iodide in an alkaline medium. In the HPLC analysis, two species were detected. The product with a retention time (rt) of 13.2 min corresponds to an unreacted glabranin, while the product with a 15.2 min rt corresponds to the methylated glabranin derivative (FLA), as confirmed by spectroscopic methods. The methylated derivative was used to verify the identity of the radiolabeled analog through coinjection. In addition, the synthesized compound was used to determine the IC_50_ of FLA to assess if methylation affects the inhibitory activity towards GSK-3β (see biological evaluation).

### 2.2. Radiochemistry

**Figure 2:**
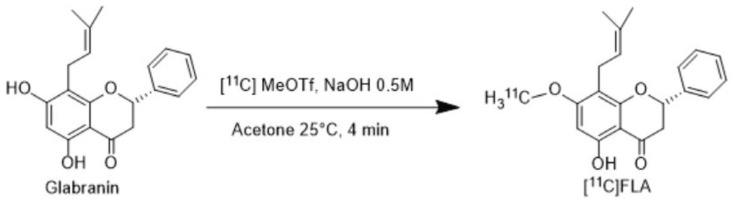
Radiosynthesis of [^11^C]FLA.

^11^C labeling of glabranin was performed by O-methylation on the OH group at position 7 of the A ring, as shown in Figure 2. The methylation position was selected due to the high reactivity of the above-mentioned hydroxyl group reported in the literature [24,25].

Different reaction conditions were assayed to maximize labeling yield, as shown in Table 1.

The initial labeling test was designed based on the working group’s prior experience, employing [^11^C]CH₃I as the methylating agent and 5 M sodium hydroxide (NaOH) as the base, at 80 °C for 1 min in dimethyl sulfoxide (DMSO) (Table 1, entry I). The radio-HPLC analytical chromatogram revealed that the desired radiolabeled product, [^11^C]FLA, was not formed under these conditions.

Subsequently, an alternative labeling strategy was explored using tetrabutylammonium fluoride (TBAF) as the base. TBAF was selected due to its reported superior performance in O-methylation reactions involving [^11^C]CH_3_I compared to NaOH (Adam et al., 2000; Ding et al. [26,27]. The labeling conditions were adjusted accordingly, with [^11^C]CH_3_I as the methylating agent, 1 M tetrahydrofuran (THF) as the solvent, and an extended reaction time of 5 min at 80 °C (Table 1, entry II), based on the protocol described by Adam et al. (2000) [26]. However, HPLC analysis indicated that [^11^C]FLA was not formed under these conditions, either.

The use of methyl iodide as a methylating agent (conditions I and II) did not yield the desired product, and, consequently, was replaced by methyl triflate (condition III), due to its greater reactivity [28,29].

The radio-HPLC analytical chromatogram of the reaction mixture showed the presence of [^11^C]methanol (rt 5.2 min) generated by the hydrolysis of the [^11^C]methyl triflate, and a main peak with an rt 15.4 min corresponding to the desired product [^11^C]FLA.

**Figure 3:**
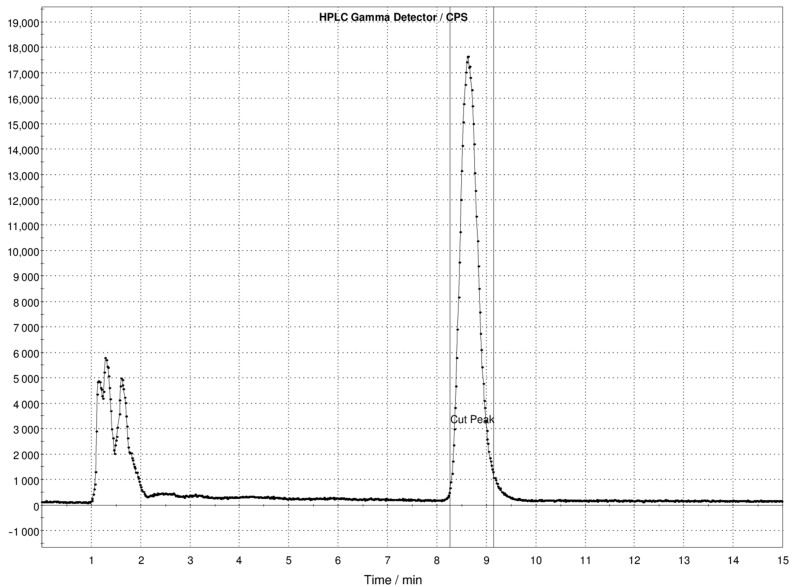
Semipreparative gamma-chromatographic profile of unpurified [^11^C]FLA.

Purification was successfully achieved by reverse-phase semi-preparative HPLC. An example of the gamma-chromatographic profile of unpurified [^11^C]FLA is shown in Figure 3.

The highest yield (16 ± 3% decay-corrected yield) was obtained using [^11^C]MeOTf as a methylating agent and 2 µL de NaOH 0.5 M in anhydrous acetone at 25 °C for 4 min (entry III, Table 1).

**Figure 4:**
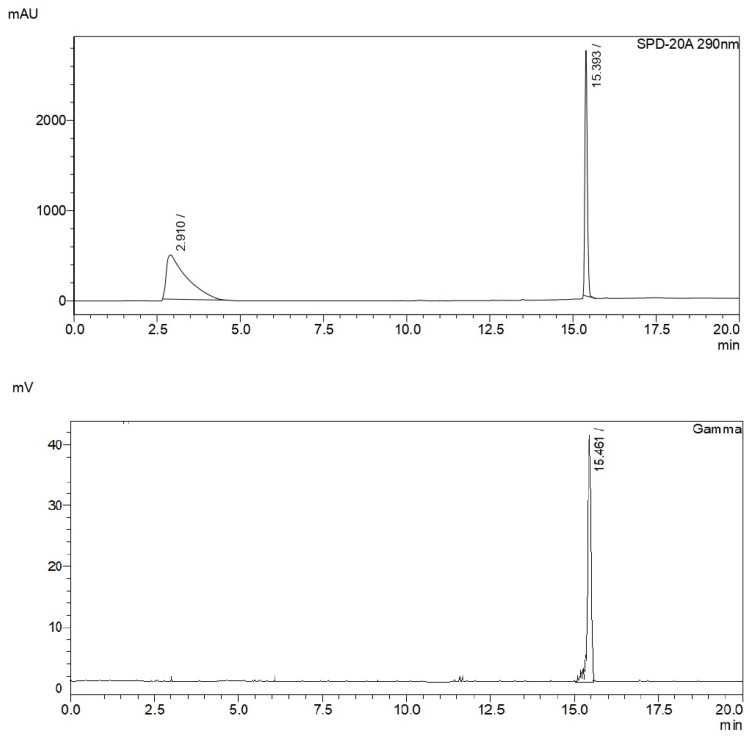
Analytical radiochromatographic profile of the coinjection of [^11^C]FLA and FLA. The upper panel shows UV detection at 290 nm corresponding to FLA, while the lower panel displays gamma detection corresponding to [^11^C]FLA.

After purification, the product was diluted with water, retained on a solid-phase extraction cartridge (C18 SepPak Light—Waters, Milford, MA, USA), and finally diluted in aqueous 0.9% NaCl with 10% ethanol. Analytical radio-HPLC chromatograms showed the presence of the desired ^11^C-methylated product as a unique product with a retention time of 15.5 min. Coincidence with the nonradioactive analogous (FLA) upon coinjection confirmed the identity of the radiolabeled product (Figure 4).

The potential methylation of the OH group in position 5 was not observed, thus confirming the literature reports about the higher reactivity of OH in position 7 in flavanones [30,31].

### 2.3. Physicochemical Evaluation

Characterization of [^11^C]FLA was performed by the following physicochemical studies: (i) stability at the final formulation; (ii) stability in plasma; (iii) plasma protein binding, and (iv) lipophilicity. These studies are predictive and could be then translated to the in vivo behavior of the radiotracer.

[^11^C]FLA was stable both in its final formulation and in human plasma for at least 150 min, with radiochemical purity (RCP) > 90%. This time frame is adequate for an ^11^C-labeled compound and sufficient to perform biological evaluations. Binding to plasmatic proteins was determined by size exclusion chromatography. PPB is a very important parameter that determines not only the pharmacokinetics of the drug but also its ability to penetrate the biological membranes [32]. The PPB of [^11^C]FLA, determined by molecular exclusion, was 72 ± 3% (n = 3) and remained constant over time (for up to 60 min). The lipophilicity, determined as the partition coefficient between n-octanol and the phosphate buffer (pH = 7.4), was LogP 7.4 = 1.22 ± 0.05 n = 3. This value is in accordance with the chemical structure of FLA and is within the optimal range to cross the BBB LogP at pH 7.4 between 0 to 3 [33].

The physicochemical studies corroborated that the molecule obtained is stable and presents adequate lipophilicity to cross membranes, which supports the proposal of this radiotracer for the experimental validation of in silico results about the interaction of these compounds with GSK-3β.

### 2.4. Ex Vivo Biodistribution Studies

**Figure 5:**
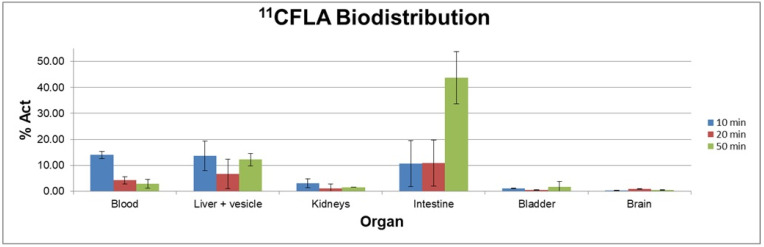
Biodistribution of radioactivity after intravenous (i.v.) injection of [^11^C]FLA in C57BL6J black mice.

Biodistribution studies with [^11^C]FLA were carried out in C57BL6J black mice at different times post-injection. Figure 5 shows the results expressed as % activity/organ in the most significant fluids and organs as a function of time.

Biodistribution studies show that [^11^C]FLA is highly cleared from the blood (13.9 ± 1.7% at 10 min post-injection, 2.8 ± 1.7% at 50 min post-injection). Liver uptake was very high, even after 50 min of biodistribution time (12.2 ± 2.4%), and excretion occurred mainly through the hepatobiliary system (% in intestines 44 ± 10 at 50 min post-injection), with only a minor fraction excreted in the urine.

**Figure 6:**
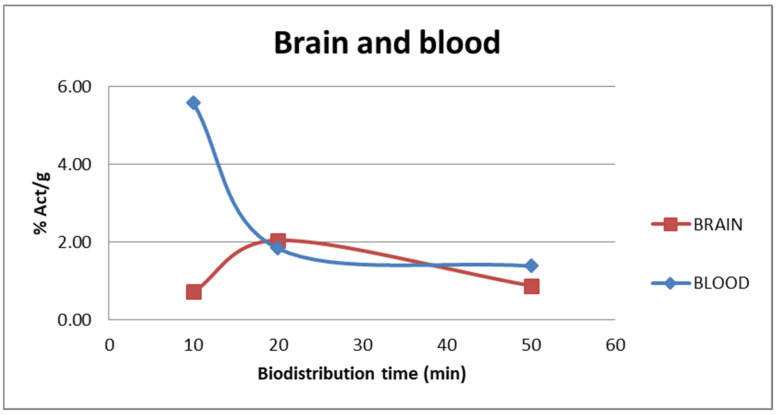
% Activity/g vs. t in blood and brain as a function of time after injection of [^11^C]FLA.

The tracer uptake in the blood and brain at different injection times of [^11^C]FLA are shown in Figure 6. At 20 min, when maximum uptake in the brain is reached (2.5 ± 0.29), clearance from the blood had already begun (5.58 ± 0.81% at 10 min post-injection, 1.84 ± 0.62% at 20 min post-injection), suggesting some retention mechanism within the brain.

### 2.5. In Vivo Imagining Studies

**Figure 7:**
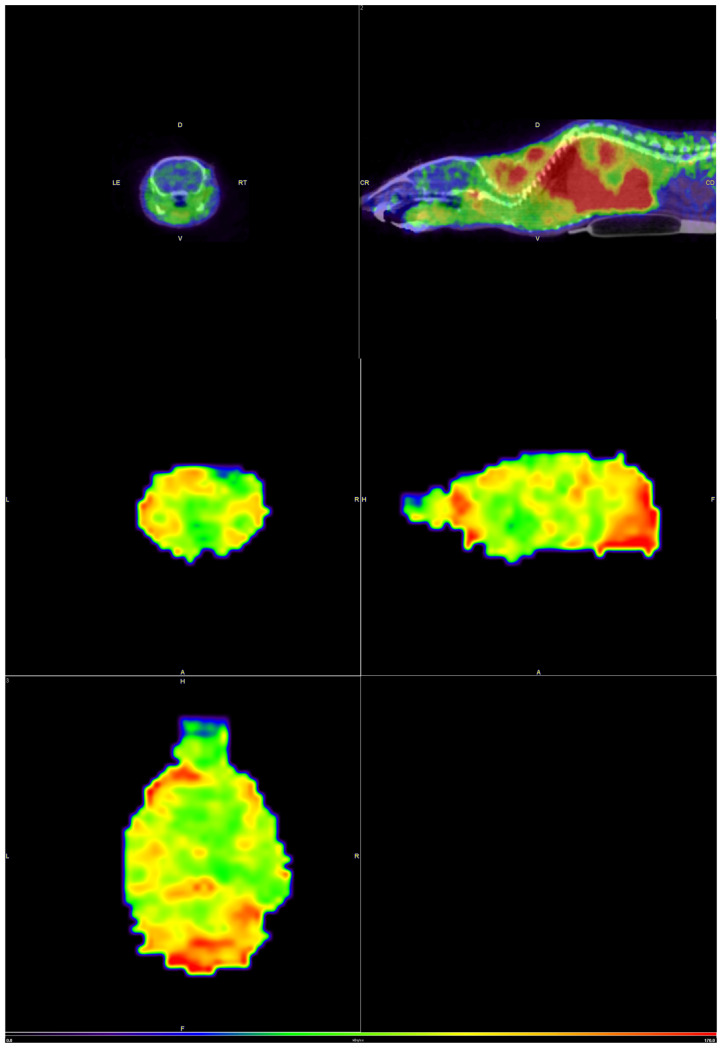
Static images (axial, sagittal, and coronal slices) at 20 min post-administration of [^11^C]FLA showing the uptake in a normal animal, in the body (**upper images**) and brain (**lower images**).

Static PET images were acquired in healthy C57BL/6J male mice, to confirm that the compound FLA is capable of crossing the blood–brain barrier (Figure 7).

Uptake was seen in the brain, as expected by the logP at pH 7.4 value of the compound, and the result is consistent with the results obtained in the bi-distributions, which are shown previously.

### 2.6. In Vitro Evaluation of GSK-3β Inhibition

**Figure 8:**
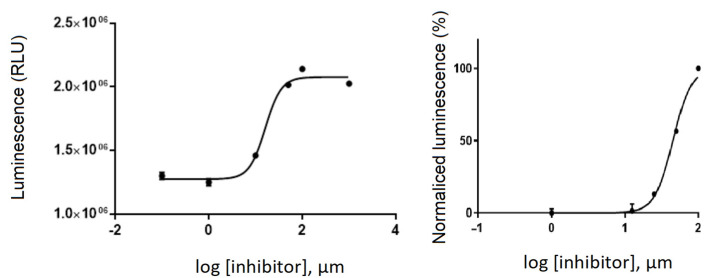
Dose–response curve, by determination IC_50_ of glabranin and FLA. The data shown are the means of three different experiments.

A luminescence method based on the quantification of the ATP amount present after the kinase reaction was employed to assess the GSK-3β inhibitory effect by glabranin and FLA. Compounds were first tested at a fixed concentration of 10 μM (in buffer), and a low GSK-3β inhibitory effect for both was observed. Then, the IC_50_ value was determined by performing a dose–response curve using different concentrations (1–100 μM). Nonlinear regression analysis, based on three independent experiments, showed an IC_50_ value in the micromolar range, being 44.8 ± 4.0 µM for glabranin, and 16.14 ± 0.6 µM for FLA (Figure 8).

This study confirmed the literature-reported inhibitory activity of GSK-3β by glabranin, and demonstrated that this activity was not eliminated by methylation. Furthermore, a lower IC_50_ of FLA indicated that methylation slightly potentiated the inhibition of the enzyme.

### 2.7. In Vitro Evaluation of [^11^C]FLA Uptake by Neurons

A primary culture of neurons from the hippocampus and cortex was used to test [^11^C]FLA uptake in vitro since these are the brain regions where GSK-3β is abundant [34]. Likewise, these are neuronal populations that are particularly affected by AD [35].

The uptake of [^11^C]FLA in cultured neurons was determined at three incubation times (5, 10, and 20 min). The uptake percentage varied significantly within the incubation time. 20 min was the time in which the highest percentage (15%) of uptake was observed (Table 2).

[^11^C]FLA uptake by neurons was also evaluated at different activity loadings of the radiolabeled product. The variation of uptake percentage with the activity added to the neuron culture is shown in Table 3. A higher binding was obtained when the cultures were incubated with a lower activity.

These results were also used to optimize the conditions to test [^11^C]FLA uptake in other cell types, such as astroglial cells. [^11^C]FLA uptake by primary cultures of astrocytes is used as a negative control since GSK-3β is primarily expressed in neurons [36].

### 2.8. In Vitro Evaluation of [^11^C]FLA Uptake by Astrocytes

The uptake of [^11^C]FLA by primary cultures of astrocytes is shown in Table 4. Compared with the uptake in neurons (Figure 2), the uptake by astrocytes was significantly lower at all studied times (5, 10, and 20 min). Furthermore, the uptake at 20 min was two-fold greater in neurons (14%) than in astrocytes (7%), suggesting the specificity of [^11^C]FLA uptake.

### 2.9. Competition Assay

[^11^C]FLA interaction with GSK-3β enzyme was evaluated.

A competition assay between [^11^C]FLA and a recognized GSK-3β inhibitor, the 3-acetyl-4-(1-methyl-1H-indol-3-yl)-1H-pyrrole-2,5-dione (VP.38) was performed to further evaluate selectivity towards GSK-3β. Perez et al. previously reported an IC_50_ value for this maleimide derivative of 0.89 ± 0.19 µM [37].

**Figure 9:**
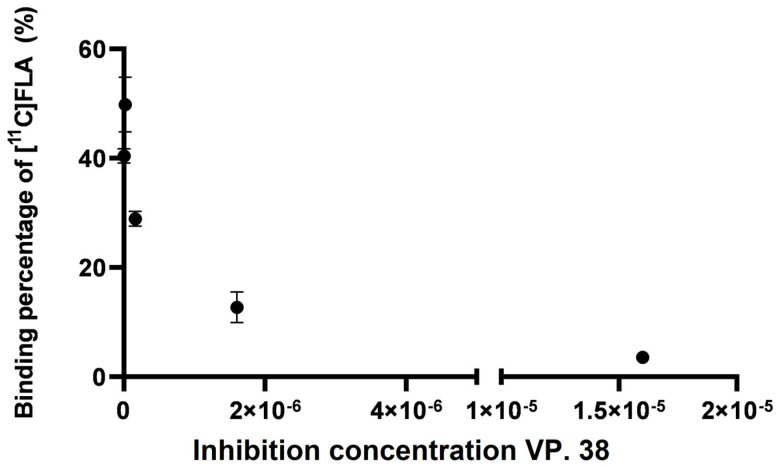
[^11^C]FLA uptake by neurons when challenged against the commercially available GSK-3β inhibitor VP.38.

The uptake of [^11^C]FLA by neurons varied significantly with VP.38 concentration, for example, 49.3 ± 8.2% with 1.6 × 10^−8^ M of VP. 38 and 3.32 ± 0.4% with 1.6 × 10^−5^ M, as shown in Figure 9. This result strongly suggests that [^11^C]FLA interacts with GSK-3β in neurons.

## 3. Discussion

Historically, natural products have played a very important role in drug discovery due to many advantages, especially the higher chemical diversity and structural complexity in comparison to synthetic molecules [38]. In recent years, there has been a growing trend toward leveraging biology-inspired chemistry strategies to design novel bioactive compounds. These approaches aim to expand the chemical space of drug discovery while maintaining the biological relevance of natural products [39,40]. The main areas of development have been cancer and infectious diseases [41,42,43], but some successful examples can also be found in cardiology (for example, statins) [44], and some neurodegenerative diseases (for example, fingolimod in multiple sclerosis) [45].

However, there are some important concerns regarding their use, mainly, the lack of scientific evidence for their efficacy and safety [46].

Positron emission tomography can contribute to overcoming this drawback, providing information about target engagement, proof of mechanism, pharmacokinetic and pharmacodynamic profiles, etc. [47]. PET is a molecular imaging technique that allows for the monitoring of a drug treatment’s effectiveness in vivo by studying the functionality of organs and biochemical processes. PET technology is very useful in the discovery and development of potential new drugs, as it allows determining whether a potential drug reaches its pharmacological target and obtaining information on whether it presents specific binding to a receptor, etc.

In the particular case of molecules targeting the central nervous system, such as glabranin, a crucial point is the determination of penetration through the blood–brain barrier and access to the site of action. These points can easily be determined qualitatively and quantitatively by using PET technology by marking the molecule to be studied with a positron-emitting radionuclide [48,49]. The advantage of positron-emitting radionuclides such as ^11^C and ^18^F is that they can be easily incorporated into a wide variety of organic compounds without altering their structure, as well as their physical, chemical and biological properties [50].

In this context, our goal was to demonstrate the interaction between glabranin, a potentially bioactive molecule derived from a native plant from our region, and GSK-3β as the molecular target described in the literature.

Our experimental design involved preparing an ^11^C derivative of glabranin through methylation. The experimental conditions were optimized, and the expected product could be isolated with an adequate yield and purity. Furthermore, [^11^C]FLA was stable both in the formulation medium (ethanol in NaCl 0.9%) and in human plasma for at least 150 min, thus allowing its use in in vitro and in vivo assays.

The structural verification through comparison with the non-radioactive analog allowed us to demonstrate that methylation occurred in the OH group in position 7, which was described in the literature as the most reactive position.

The physicochemical evaluation demonstrated that the lipophilicity of [^11^C]FLA was within the optimal range for crossing the blood–brain barrier, and that it is, consequently, potentially able to reach its cellular target. The ability of [^11^C]FLA to cross the BBB was confirmed by biodistribution results and the PET image, in accordance with previous work with different flavonoids [51,52,53].

The biodistribution profile characterized by high hepatobiliary elimination and low uptake in non-target tissues, such as the heart, spleen, and muscles, at all analyzed times, is consistent with the high lipophilicity and PPB demonstrated by physicochemical evaluation, and reflects the absence of non-specific binding, except in the lungs, as reported for other flavonoids [54]. It is also important to note that the initial uptake of [^11^C]FLA in the blood was low and decreased significantly throughout the study, denoting the tracer’s rapid blood clearance.

Additionally, we were able to verify that the compound crosses the blood–brain barrier, achieving its maximum uptake at 20 min. It is also important to note that when the maximum uptake in the brain is reached, the clearance from blood is already a significant indication that some retention mechanism inside the brain is in place.

To validate the use of the methylated analogue in the biological in vitro studies, the IC_50_ of glabranin and the methylated derivative were determined, obtaining similar values in the micromolar range for both compounds (44.8 ± 4.0 µM and 16.1 ± 0.6 µM, respectively). Although these values are higher than the ones reported in the literature for other synthetic GSK-3β inhibitors (for example, [^11^C]Tideglusib IC_50_ = 60 nM [55]), these are, to the best of our knowledge, the first reported IC_50_ values for these types of compounds. Furthermore, the objective of elucidating whether glabranin and similar prenylated flavanones effectively interact with GSK-3β was fulfilled, as reported by in silico studies.

Additional in vitro studies were included to further substantiate our hypothesis. Uptake of [^11^C]FLA by neurons from the hippocampus and cortex of mice (areas most affected in AD [35], and in which GSK-3β is most abundant) was compared with uptake in astrocytes (negative control). A high binding was observed in neuronal cultures, increasing with lower seeding activity and longer incubation time. It was also significantly higher than in the astrocyte cultures at all times tested (5, 10, and 20), being twice as high at 20 min—an expected result since GSK-3β is more abundant in neurons than in astrocytes.

The competition assay using a GSK-3 β inhibitor of proven activity (VP.38, IC_50_ 0.89 ± 0.19 µM [37]), showed a significant decrease in uptake, suggesting the specificity of the interaction with the enzyme. This assay corroborated experimentally the predictions of the in silico studies in the complex cellular environment where multiple factors are intervening. In the future, trials will be conducted to study what type of interaction occurs between GSK3 and the compound, and whether it involves a reversible inhibition, as theorized in the in silico trials.

## 4. Materials and Methods

### 4.1. Chemistry

#### Synthesis of (S)-5-hidroxy-7-methoxy-8-(3-methylbut-2-en-1-yl)-2-phenylcroman-4-one (FLA)

(S)-5-hidroxy-7-methoxy-8-(3-methylbut-2-en-1-yl)-2-phenylcroman-4-one (FLA) was obtained by the direct methylation of glabranin (50 mg, 0.15 mmol) with methyl iodide (0.200 mL, 3.22 mmol), in basic conditions (K_2_CO_3_ (0.432 g, 3.13 mmol) in acetone (5 mL), at room temperature and in an N_2_ atmosphere. The reaction progress was followed by analytical HPLC using a reverse-phase Agilent ZORBAX Eclipse Plus column (Agilent) C18 4.6 × 150 mm 5 μm, a flow rate of 1.0 mL/min, and the following solvent gradient: (A) acetic acid 0.1% (*v*/*v*) in water, (B) acetic acid 0.01% (*v*/*v*) in acetonitrile; 0 min: 10% (B), 0–10 min: 10 to 100% (B), 10–20 min: 100% (B). After 60 min of reaction, the chromatogram showed two products with retention times (rt) of 13.2 and 15.2 min, respectively. The products were isolated by analytical HPLC and identified using NMR spectroscopy. FLA: ^1^H NMR (CDCl_3_) δ 12.13 (s, 1H), 7.47–7.37 (m, 5H), 6.10 (s, 1H), 5.43 (dd, 1H, j = 3.12), 5.14 (t, 1H, j = 8), 3.25 (d, 2H, j = 7), 3.09–3.01 (m, 1H), 2.83 (dd, 1H, j = 3.17), 1.65(d, 6H, j = 12) ^13^C NMR (CDCl_3_) δ 196.2 (C 4), 166.7 (C 7), 162.9 (C 5), 138.9 (C 1′), 128.7 (C 5′), 128.5 (C 4′), 125.9 (C 6′), 122.4 (C 2″), 108.9 (C 8), 102.9 (C 10), 92.5 (C 6), 78.6 (C 2), 55.9 (C 1‴), 43.4 (C 3a), 25.7 (C 4″), 21.6 (C 1′), 17.6 (C 5″).

### 4.2. Radiochemistry

Radiosynthesis of (S)-5-hydroxy-7-^11^C-methoxy-8-(3-methylbut-2-en-1-yl)-2-phenylchroman-4-one ([^11^C]FLA). Radiosynthesis was carried out in the synthesis module TRACERlabFXCPro (GE Healthcare, Chicago, IL, USA).

Three different labeling conditions were tested, which differed in methylating agent (methyl iodide [^11^C]CH_3_I or methyl triflate [^11^C]CH_3_OTf), base (NaOH, TBAF), time (1, 4, and 5 min), temperature (25 and 80 °C), and solvent (DMSO and acetone), were assayed for the preparation of [^11^C]FLA

[^11^C]CH_3_I and [^11^C]CH_3_OTf were prepared according to the previously described method by P. Buccino et al. [56].

The optimized procedure was performed as follows: [^11^C]CH_3_OTf was bubbled into the reactor containing the precursor (S)-5,7-dihidroxy-8-(3-methylbut-2-en-1-yl)-2-phenylcroman-4-ona (0.2 mg, 0.745nmol) dissolved in anhydrous acetone (300 µL) and 2 µL de NaOH 0.5 M. Once the radioactivity was fully transferred, the reaction mixture was incubated at 25 °C for 4 min. The reaction mixture was diluted with the acetonitrile/water (30:70) (1.5 mL) and purified onto a reverse-phase semipreparative VP 125/10 NUCLEODUR C18 HTec (Macherey–Nagel, Düren, Germany) 5 μm column with an isocratic mixture of deionized water and acetonitrile (70:30) and a flow rate of 8 mL/min. The product was collected, diluted with 50 mL of deionized water, and loaded in a solid-phase extraction cartridge (C18 SepPak Light—Waters, Milford, MA, USA) to remove excess acetonitrile. The product was eluted from the cartridge with absolute ethanol (1.0 mL) and further diluted with saline (10% ethanol in saline). The final product [^11^C]FLA was collected through a sterile 0.22 μm pyrogen-free filter (Millex SA, Cordoba, Argentina), into a sterile vial. The radiochemical purity of [^11^C]FLA was determined using the above-described HPLC system. Confirmation of the product identity was performed by the coinjection of the nonradioactive FLA.

### 4.3. Physicochemical Evaluation

Stability in Formulation Milieu (10% ethanol in NaCl 0.9%). Isolated [^11^C]FLA was evaluated at 3 h after labeling. The RCP purity was determined using by the method described Fernandez et al. [57].

Stability in Plasma. [^11^C]FLA (100 μL) in human plasma was assessed at 60 and 120 min incubation time, as described by Giglio et al. [58].

Lipophilicity. Lipophilicity was studied through the partition coefficient (P) between n-octanol and phosphate buffer 0.125 M, pH 7.4, as described by Soledad Fernandez et al. [59]. Lipophilicity was expressed as LogP, where P was calculated as the mean value (cpm/mL) of the n-octanol layer divided by that of the buffer.

Protein Binding (PPB). [^11^C]FLA PPB radiotracer was calculated as the percentage of activity eluted from the column Microspin G-50 columns (Pharmacia Biotech, Amersham, UK), following the method described by Cardoso et al. [60].

### 4.4. Biological Evaluation

C57BL/6J male mice, 13–14 weeks old, were used for ex vivo and in vivo studies. The animals were housed under 12:12 h light/dark cycles; food and water were given ad libitum in rooms with controlled temperature and humidity at the Uruguayan Centre of Molecular Imaging (CUDIM) animal facility. The research protocol was carried out in accordance with the National Bioethics Committee requirements and under the current ethical regulations of the national law on animal experimentation N° 24042301 (National Commission of Animal Experimentation, CNEA, Montevideo, Uruguay). CUDIM’s Animal Bioethics Committee approved these protocols.

### 4.5. Ex Vivo Biodistribution Studies

Biodistribution studies were performed in C57BL/6J male mice, weighing between (36 ± 3) g (n = 8). The animals were injected i.v. with 0.8–12.6 MBq (200–250 µL) of [^11^C]FLA and sacrificed by cervical dislocation at 10, 20, and 50 min after injection (n = 3 animals in each injection time). Organs (intestine, liver, kidneys, bladder, and brain) and blood samples were extracted and weighed, and their activity was measured in a gamma counter (a 300 × 300 well-type NaI [Tl] solid scintillation detector coupled to a multichannel analyzer, ORTEC). The biodistributions studies were performed following the method described by Kreirmerman et al.

### 4.6. In Vivo Imaging Studies

Small-animal PET-CT imaging was performed in a nanoScan^®^ PET-CT Mediso Preclinical Imaging system, based on LYSO scintillators. The spatial resolution of the scanner is 0.9 mm, and the transaxial field of view (FOV) is 10.0 cm. The data were acquired in list mode in a 212 × 212 × 235 matrix with a pixel size of 0.4 × 0.4 × 0.4 mm and a coincidence window width of 1.0 nsec. 2% isofluorane in an oxygen flow of 2 L/min is used to anesthetize the mice. The animals injected i.v. via the caudal tail vein with 200–300 μL of [^11^C]FLA (10.1 ± 2.3 MBq). PET image (static study) acquisition started 15 min after radiotracer administration and performed over 20 min. Sinograms were reconstructed using 3D maximum likelihood expectation maximization (3D-MLEM) with 4 iterations and 6 subsets.

### 4.7. In Vitro GSK-3β Inhibition Assay

Human recombinant GSK-3β and GSK3 substrate (YRRAAVPPSPSLSRHSSPHQ(pS)EDEEE) were purchased from Promega (GSK-3β Kinase Enzyme System, Ref. V1991). Kinase-Glo luminescent kinase assay was obtained from Promega (Promega Biotech Iberica, Madrid, Spain, SL, Ref. V6711). ATP and all other reagents were from Sigma-Aldrich (St. Louis, MO, USA). The assay buffer contained 50 mM HEPES (pH 7.5), 1 mM EDTA, 1 mM EGTA, and 15 mM magnesium acetate. The GSK-3β activity assay was performed following the method published by Baki et al. [61]. The enzymatic reaction was performed in assay buffer (total volume, 40 μL) in white 96-well plates. In a typical assay, 10 μL of FLA or glabranin (dissolved in DMSO at 10 mM concentration and then diluted in assay buffer to the desired concentration), and 10 μL (22 ng) of enzyme were added to each well, followed by 20 μL of assay buffer containing substrate (GS-2 peptide) and ATP, the final concentrations in the well being 25 μM and 1 μM, respectively. The final DMSO concentration in the reaction mixture did not exceed 1%. After a 30 min incubation period at room temperature, the enzymatic reaction was stopped with 40 μL of Kinase-Glo reagent. Glow-type luminescence was recorded after 10 min using a GloMax^®^ Discover Microplate Reader(Promega, Madison, WI, USA). The activity is proportional to the difference between the total and consumed ATP. The inhibitory activities were calculated based on maximal activities measured in the absence of an inhibitor. The IC_50_ was defined as the concentration of each compound that reduced by 50% the enzymatic activity compared to that without inhibitors. The IC_50_ value was determined by performing a nonlinear regression analysis using five different concentrations. Curve fitting was performed using the sigmoidal dose–response function on GraphPad Prism 6.0 software. The data shown are the mean of three different experiments.

### 4.8. Neurons and Astrocytes Primary Cultures

A primary culture of hippocampal and cortical neurons was prepared from 16–18-day-old fetuses of C57BL/6J mice, as previously described.

Cells were cultured in a monolayer in 6-well plates at a cell density of 5 × 10^5^ cells/well and were maintained in a neurobasal medium supplemented with 2% B27, 1% Glutamax, and penicillin/streptomycin (1%) at 37 °C and 5% CO_2_. The culture medium was partially changed every other day until the moment of the trials.

A culture of astrocytes from the hippocampus and cortex of neonatal C57BL/6J mice (1–2 days old) was prepared according to the method described by Diaz-Amarilla et al. [62]. Cells were cultured in a monolayer on individual P6 plates and maintained in DMEM medium supplemented with penicillin/streptomycin (1%) in a humidified incubator, at 37 °C and 5% CO_2_.

### 4.9. Uptake Studies in Neurons and Astrocytes

After 12 days in vitro, the neurons or astrocytes were incubated with 14 µCi (200 µL) of [^11^C]FLA for 5, 10, and 20 min, and with 14 µCi, 27 µCi and 54 µCi of the radiolabelled product for 20 min (assays were performed in triplicate) at 37 °C and 5% CO_2_. After the incubation time, the culture medium was preserved, and cells were washed with 1 mL phosphate-buffered saline (PBS). The cells were then detached with 500 µL trypsin-EDTA followed by 5 min of incubation at 37 °C and 5% CO_2_, and the radioactivity of the culture medium and the radioactivity bound to the cells were measured in a solid scintillation counter. The uptake percentage was expressed as the percentage of radioactivity bound to cells relative to total activity (medium radioactivity + cell-bound radioactivity), as is shown in the following equation:

cell-bound radioactivity/(medium radioactivity + cell-bound radioactivity) × 100

### 4.10. Competition Assay

The neurons were incubated at 37 °C and 5% CO_2_ for 20 min, with 14 µCi (200 µL) of [^11^C]FLA and increasing concentrations (16–1600 nM) of the commercially available GSK3β inhibitor, 3-acetyl-4-(1-methyl-1H- indol-3-yl)-1H-pyrrole-2,5-dione (VP.38). The cells were examined under a microscope after the addition of VP.38 under the same experimental conditions to confirm that they remained viable. After the incubation time, the culture medium was preserved, and the cells were washed with PBS and detached with trypsin-EDTA, as described above, for uptake studies. The activity of the culture medium and the activity bound to the cells were measured, and the percentage of uptake was calculated as described for uptake studies.

## 5. Conclusions

[^11^C]FLA, a methylated derivative from the natural compound glabranin, whose GSK-3β inhibitory activity was previously assessed by in silico studies, was synthesized and evaluated for the first time. Furthermore, the IC_50_ of glabranin was determined by our group, confirming the in silico prediction. Labeling, carried out with ^11^C-methylation, was optimized. The purification process developed rendered a high radiochemical purity product with a reasonable yield.

[^11^C]FLA was stable over time, and lipophilic, as expected, with an adequate value to cross biological membranes, including the BBB, a capacity confirmed in animal studies since uptake of [^11^C]FLA was seen in the brain.

In vitro studies showed a significant uptake of the radiotracer by neurons in culture, being greater than that of astrocytes. This behavior reinforces the hypothesis of the specific interaction of [^11^C]FLA with GSK-3β since this enzyme is expressed mainly in neurons.

The methodology employed, obtaining and characterizing an ^11^C-labeled methylated derivative, allowed us to easily perform the in vitro evaluation of a molecule with biological activity obtained from a native South American species. These results demonstrate the utility of PET technology in confirming the biological activity of natural products and their potential for drug discovery and evaluation.

## Figures and Tables

**Table 1:** Summary of glabranin labeling conditions.

	Methylating Agent	Base	Solvent	Temperature (°C)	Time (min)	Yield (%)
I	[^11^C]CH_3_I	NaOH 5 M 5 µL	DMSO 0.5 mL	80	1	0
II	[^11^C]CH_3_I	TBAF 1 M 100 µL	Acetone 0.4 mL and THF 0.15 mL	80	5	0
III	[^11^C]CH_3_OTf	NaOH 0.5 M 2 µL	Acetone 0.3 mL	25	4	16 ± 3 (n = 5)

**Table 2:** Uptake of [^11^C]FLA by neurons vs. incubation time.

Time (min)	Binding Percentage (%)
5	4.2 ± 0.7
10	8.4 ± 1.1
20	13.7 ± 1.5

**Table 3:** Uptake ^11^C-FLA in neurons vs. activity loaded.

Activity (MBq)	Binding Percentage (%)
0.5	8.3 ± 0.1
1	6.5 ± 0.3
2	5.4 ± 0.1

**Table 4:** Uptake [^11^C]FLA by astrocytes vs. incubation time.

**Time (min)**	**Binding Percentage (%)**
5	0.8 ± 0.1
10	1.0 ± 0.2
20	6.4 ± 0.2

## Data Availability

Data are contained within the article and Supplementary Materials.

## Supplementary Materials

The following supporting information can be downloaded at: https://www.mdpi.com/article/10.3390/molecules30040874/s1, Figure S1: ^1^H-NMR of FLA; Figure S2: ^13^C-NMR of FLA; Figure S3: COSY of FLA; Figure S4: HSQC of FLA; Figure S5: HMBC of FLA; Figure S6. Analytical gamma-chromatographic profile of unpurified [^11^C]FLA.
