# Supplementary figures and images for: 11C-Labeling of a Flavanone Extracted from a South American Native Species for Evaluation of Its Interaction with GSK-3β
Source: Molecules. 2025 Feb 14;30(4):874. doi: 10.3390/molecules30040874 (PMC11857971; doi:10.3390/molecules30040874)

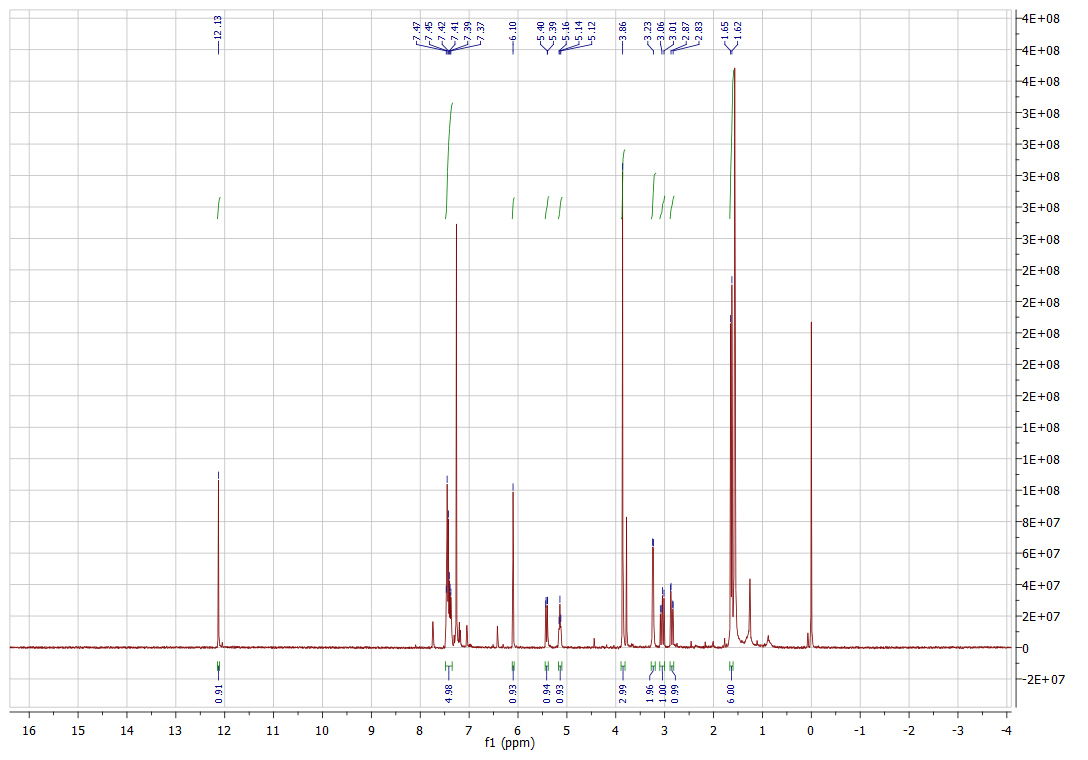

Supplement: Supplementary file 1 [file molecules-30-00874-s001.zip › Figure S1.png]

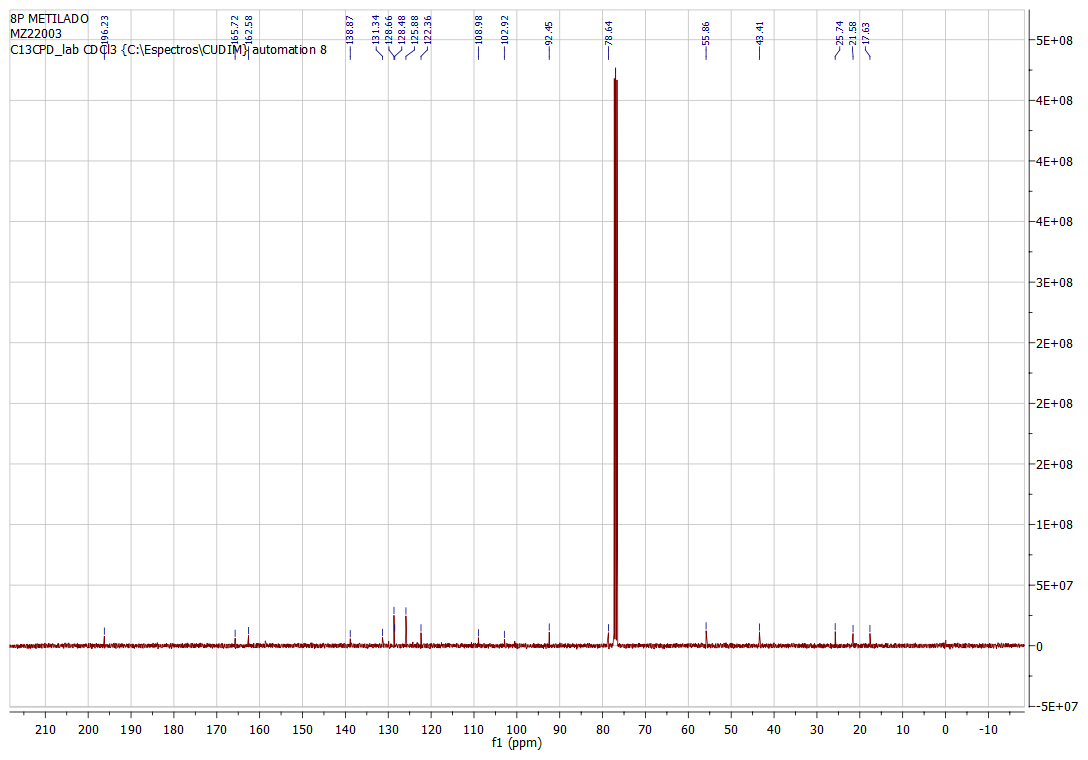

Supplement: Supplementary file 1 [file molecules-30-00874-s001.zip › Figure S2.png]

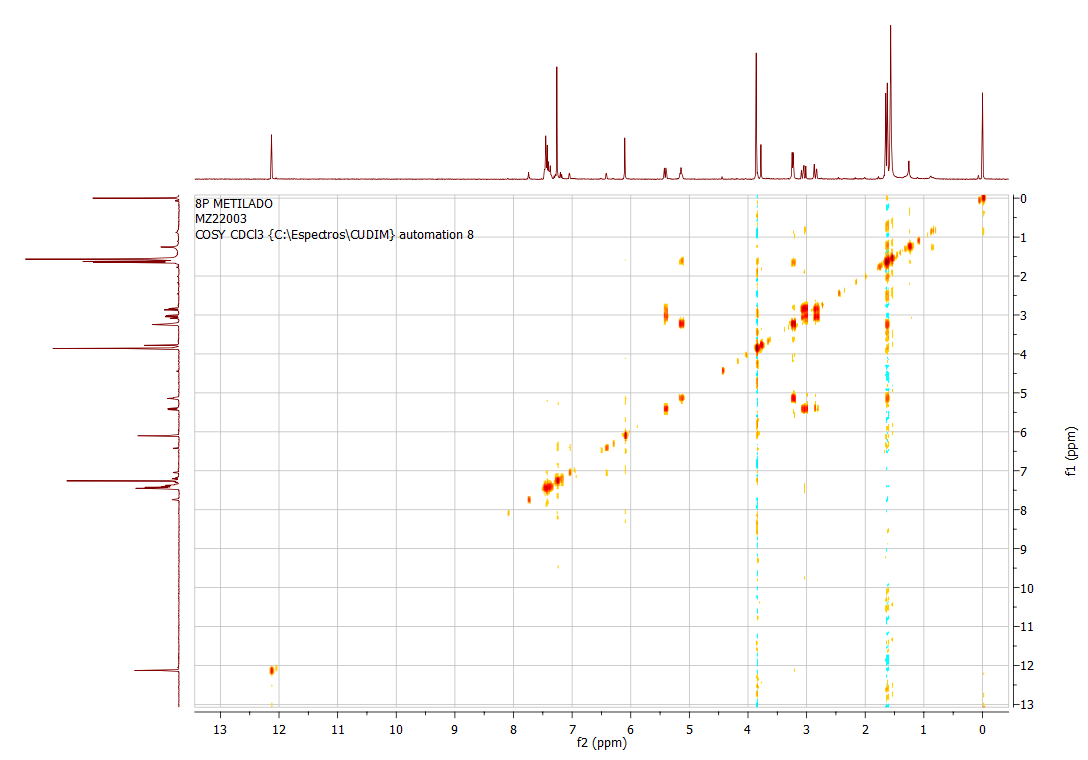

Supplement: Supplementary file 1 [file molecules-30-00874-s001.zip › Figure S3.png]

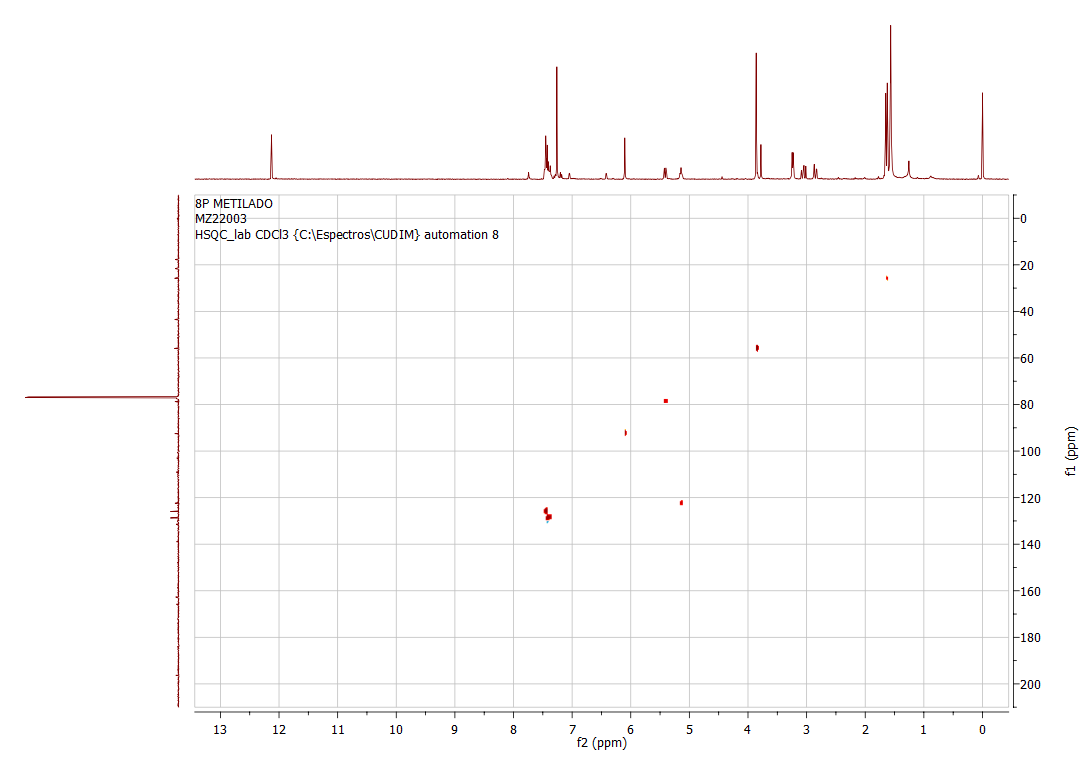

Supplement: Supplementary file 1 [file molecules-30-00874-s001.zip › Figure S4.png]

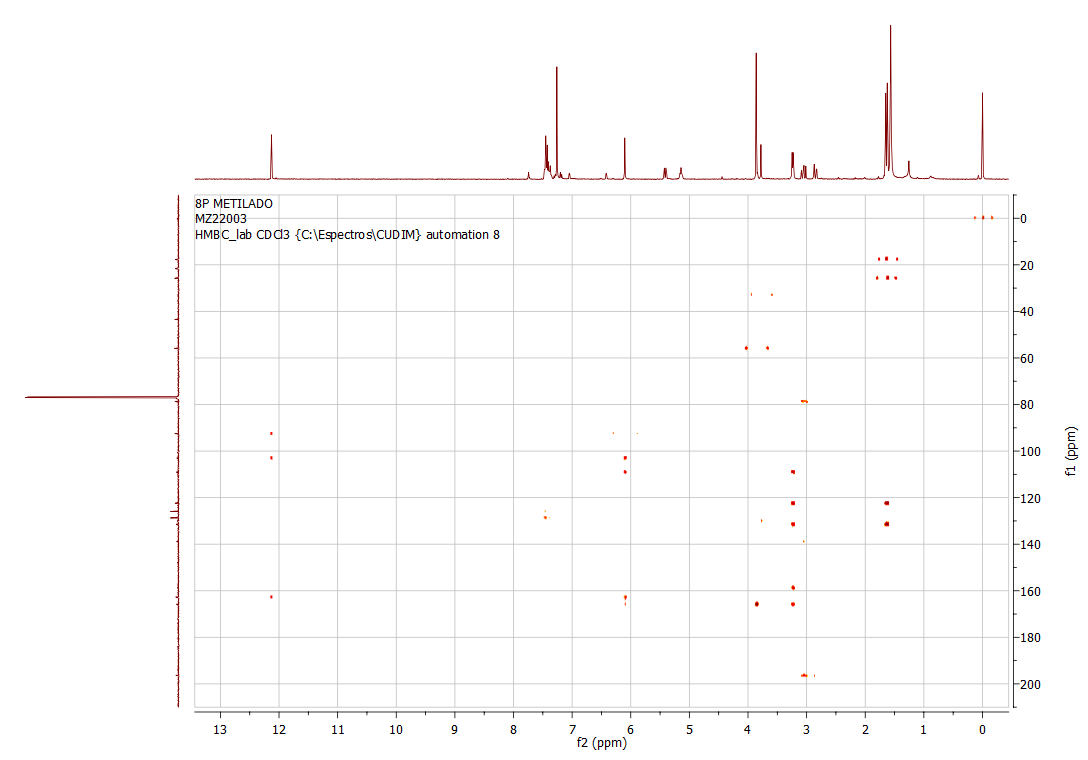

Supplement: Supplementary file 1 [file molecules-30-00874-s001.zip › Figure S5.png]

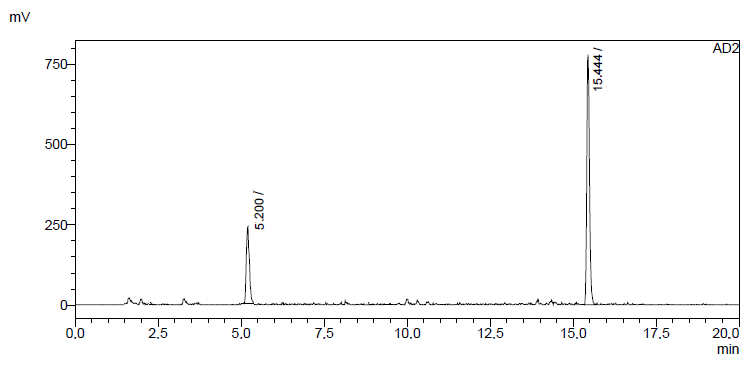

Supplement: Supplementary file 1 [file molecules-30-00874-s001.zip › Figure S6.png]
